# VEGF expression correlates with neuronal differentiation and predicts a favorable prognosis in patients with neuroblastoma

**DOI:** 10.1038/s41598-017-11637-8

**Published:** 2017-09-11

**Authors:** Wen-Chin Weng, Kuan-Hung Lin, Pei-Yi Wu, Ya-Hsuan Ho, Yen-Lin Liu, Bo-Jeng Wang, Chien-Chin Chen, Yueh-Chien Lin, Yung-Feng Liao, Wang-Tso Lee, Wen-Ming Hsu, Hsinyu Lee

**Affiliations:** 1 Department of Pediatrics, National Taiwan University Hospital and National Taiwan University, College of Medicine, Taipei, Taiwan; 2 Department of Life Science, National Taiwan University, Taipei, Taiwan; 3 Institute of Cellular and Organismic Biology, Academia Sinica, Taipei, Taiwan; 4 Department of Pediatrics, Taipei Medical University Hospital, Taipei, Taiwan; 5 Chia-Yi Christian Hospital, Chiayi, Taiwan; 6 Department of Cosmetic Science, Chia Nan University of Pharmacy & Science, Chiayi, Taiwan; 7 Department of Surgery, National Taiwan University Hospital and National Taiwan University, College of Medicine, Taipei, Taiwan; 8 Department of Electrical Engineering, National Taiwan University, Taipei, Taiwan; 9 Angiogenesis Research Center, National Taiwan University, Taipei, Taiwan; 10 Research Center for Developmental Biology and Regenerative Medicine, National Taiwan University, Taipei, Taiwan; 11 Center for Biotechnology, National Taiwan University, Taipei, Taiwan

## Abstract

Neuroblastoma (NB) is a childhood cancer with a low survival rate and great metastatic potential. Vascular endothelial growth factor (VEGF), an angiogenesis factor, has been found to be involved in CRT-related neuronal differentiation of NB cells. In this study, we further confirmed the role VEGF in NB through mouse xenograft model and clinical analysis from NB patients. In xenograft experiments, CRT overexpression effectively inhibited the tumor growth. In addition, the mRNA and protein levels of VEGF and differentiation marker GAP-43 were upregulated by induced CRT expression. However, no significant correlation between the expression level of VEGF and microvessel density was observed in human NB tumors, suggesting a novel mechanism of VEGF participating in NB tumorigenesis through an angiogenesis-independent pathway. In NB patients’ samples, mRNA expression levels of CRT and VEGF were positively correlated. Furthermore, positive VEGF expression by immunostaining of NB tumors was found to correlate well with histological grade of differentiation and predicted a favorable prognosis. In conclusion, our findings suggest that VEGF is a favorable prognostic factor of NB and might affect NB tumor behavior through CRT-driven neuronal differentiation rather than angiogenesis that might shed light on a novel therapeutic strategy to improve the outcome of NB.

## Introduction

Neuroblastoma (NB) is the most frequently diagnosed malignancy in infancy and the second most common extracranial solid tumor in childhood in Taiwan^1, 2^. It is derived from the sympatho-adrenal progenitor cells of the neural crest^3^. Children with NB exhibit a heterogeneous clinical course, from a favorable outcome with spontaneous differentiation into mature cells or regression of tumors to a poor prognosis with highly metastatic and undifferentiated tumors^3^. Although the overall outcome of NB patients has improved noticeably with recent therapeutic advances, approximately half of NB patients classified as high-risk group remain a poor prognosis with long-term survival rates no more than 40%^3, 4^. Recognizing new prognostic factors is therefore important for better understanding NB pathogenesis and developing tailored therapies that improve outcomes for NB patients.

Calreticulin (CRT) is an important chaperone protein primarily localized to the endoplasmic reticulum and highly conserved across species^5^. The multi-functional roles of CRT in protein chaperoning, Ca^2+^ homeostasis, modulating cell adhesion and regulating mRNA instability unveils its major involvement in various biological and pathologic processes^5, 6^. Accumulated evidence indicated that CRT plays an important role in the biology of NB. Previous studies reveal that increased CRT expression is correlated with better prognosis and differentiated histologies in NB^7, 8^. In addition, cell surface CRT has been found to be crucial for neurite formation when NB cells are induced to differentiate^9^. In our previous study, we found that CRT could enhance cell differentiation and suppress cell proliferation in NB cells^10^. However, how CRT affects the differentiation of NB remains unclear.

Vascular endothelial growth factor (VEGF)-A (also referred to as VEGF), a key regulator of physiologic and pathologic angiogenesis, has been reported to not only participate in the behavior of NB, but also be regulated by CRT in gastric cancer^11–13^. We have shown that CRT could positively regulate VEGF protein expression and secretion levels in condition media of various NB cell lines^10^, and the evidence that blockage of VEGF signaling could suppress neuronal differentiation in CRT-overexpressed NB cells, indicates that VEGF could be involved in CRT-regulated neuronal differentiation and might predict a favorable tumor behavior in NB. Although VEGF-driven angiogenesis has been shown to play a critical role in the pathogenesis of NB formation and metastasis^14, 15^, various studies demonstrate conflicting results regarding the role of VEGF in the tumor behavior of NB^11, 12, 16–19^.

To better understand the role of VEGF expression in the angiogenesis, neuronal differentiation, as well as tumor behavior in NB, we investigated the expression of VEGF in human NB tumors, mouse xenografts, and NB cells. The results were compared to angiogenesis and neuronal differentiation markers as well as the clinicopathological characteristics of NB.

## Results

### VEGF expression was positively correlated with CRT expression and other neuronal differentiation markers in human NB tumors, xenografts, and cells

**Figure 1:**
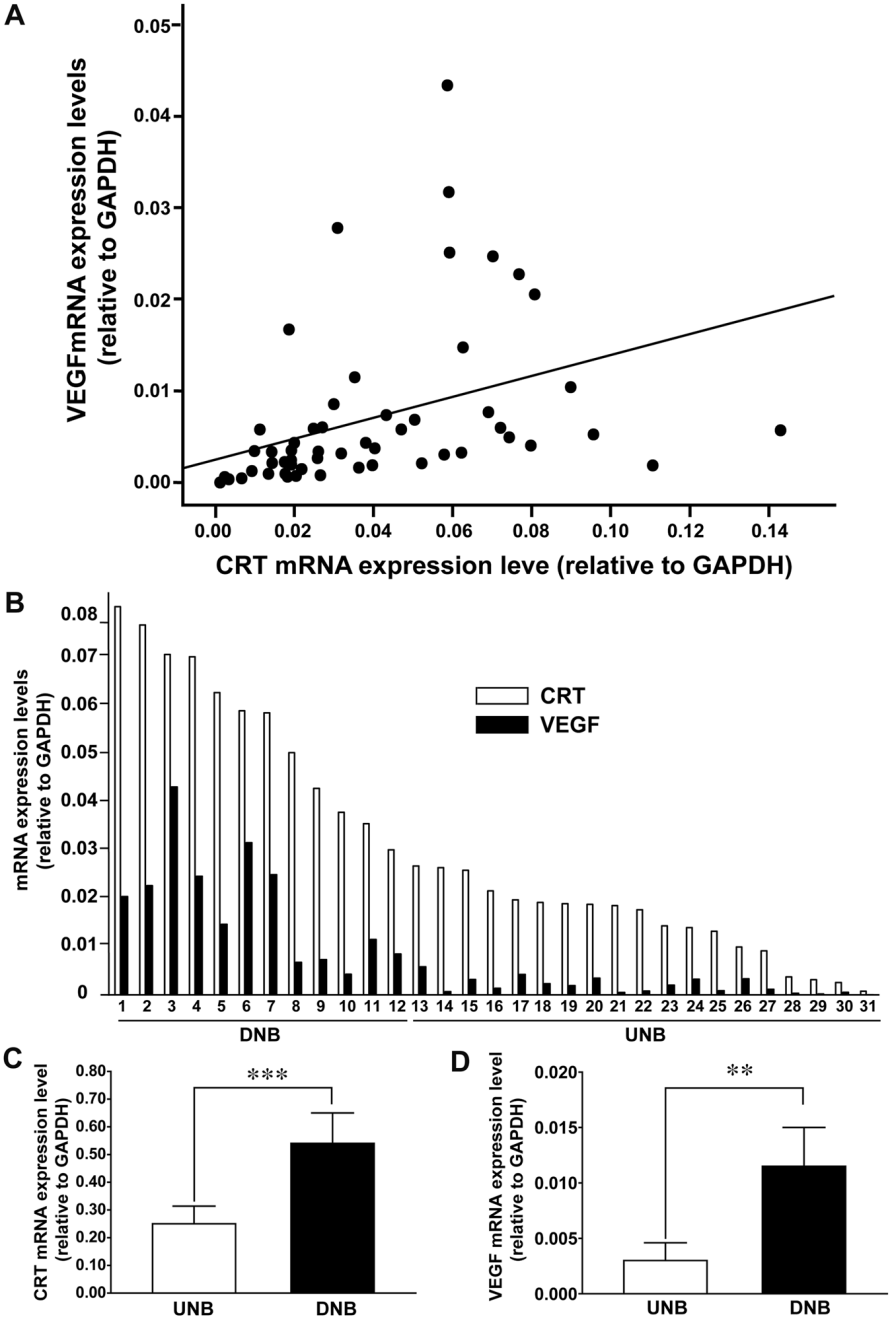
VEGF expression was positively correlated with CRT expression and differentiated histology in human NB tumors. (**A**) CRT and VEGF mRNA expressions in 56 human NB tumors were determined by real-time PCR and normalized to the internal control GAPDH. The correlation between expression levels of CRT (x axis) and VEGF (y axis) was analyzed by Spearman’s correlation test (Spearman’s ρ = 0.648, *P* < 0.001). (**B**) CRT and VEGF mRNA levels in 31 NB tumors were evaluated by real-time PCR. The levels of CRT (shaded bar) and VEGF (solid bar) are higher in differentiated NB (DNB) than in undifferentiated NB (UNB). (**C**) The CRT mRNA expression was determined by real-time PCR in 56 human NB tumors and presented as the mean ± SEM of each group, undifferentiated NB (N = 27) versus differentiated NB (N = 29). The CRT mRNA expression was significantly increased in differentiated NB. (**D**) The VEGF mRNA expression was determined by real-time PCR in 56 human NB tumors and presented as the mean ± SEM of each group, UNB (N = 27) versus DNB (N = 29). The VEGF mRNA expression was significantly increased in differentiated NB. **P < 0.01, ***P < 0.005.

Our previous studies have demonstrated that CRT may upregulate VEGF expression in NB cells. In addition, constitutive over-expression of CRT could lead to NB cell differentiation with suppressed cell proliferation^10^. To further clarify the role of CRT and VEGF expression in human NB, the mRNA expression levels of CRT and VEGF in 56 primary NB tumors were evaluated by real-time PCR. The results revealed a significantly positive correlation between CRT and VEGF expression in NB tumor tissues (Fig. 1A, Spearman’s ρ = 0.648, *P* < 0.001). Furthermore, NB with differentiated histology exhibited higher mRNA expression levels of CRT and VEGF than NB with undifferentiated histology (Fig. 1B–D).

**Figure 2:**
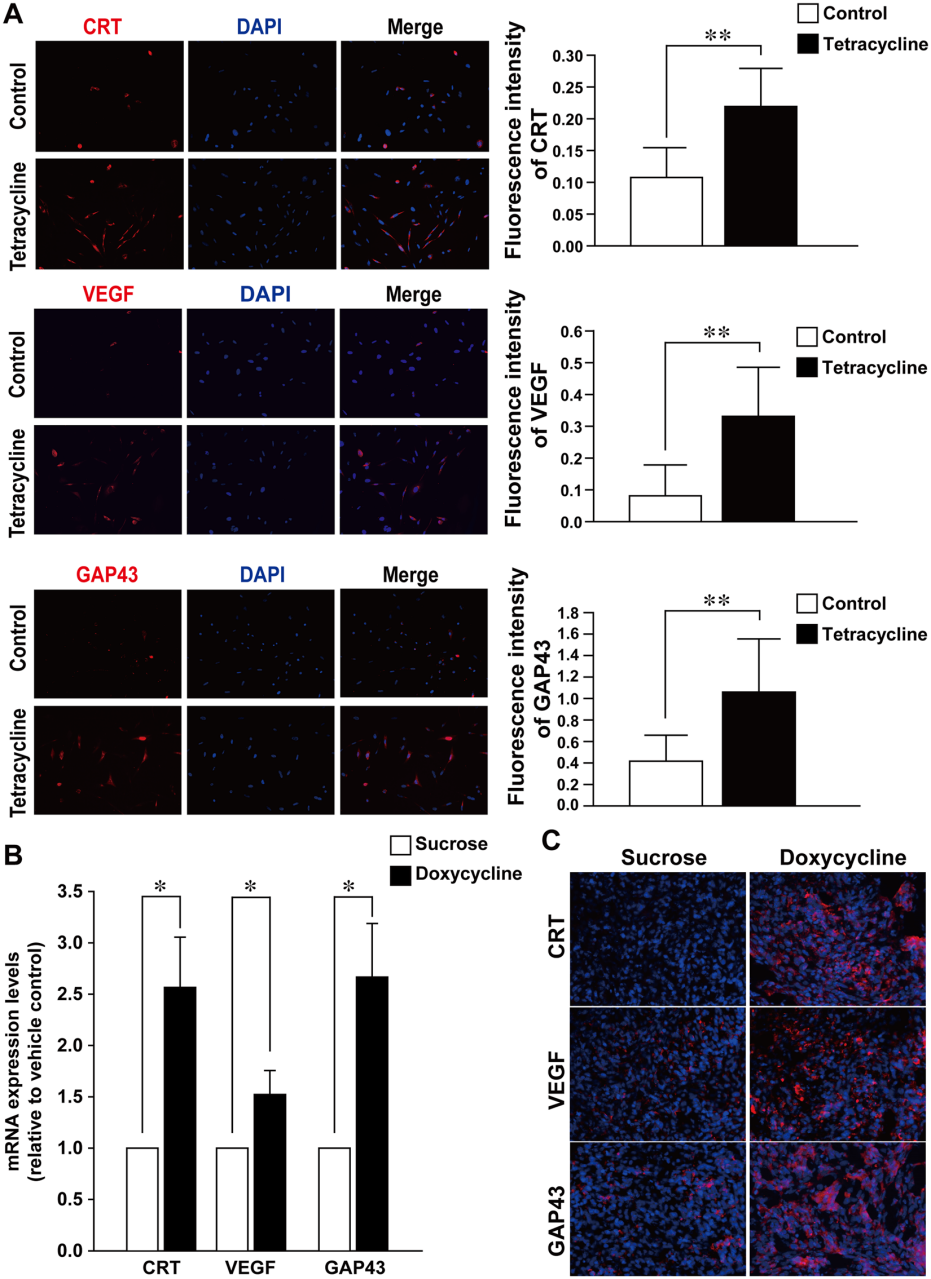
VEGF expression was positively correlated with CRT expression and neuronal differentiation in NB cells and NB xenografts. (**A**) Human stNB-V1 NB cells were stimulated with 1 μg/ml tetracycline to induce CRT expression. Expression of CRT, VEGF and GAP43 was visualized and quantitated by immunofluorescence microscopy and ImageJ. Data are shown as mean fluorescence intensity (±SD) from at least three independent experiments. (**B**,**C**) Inducible-CRT stNB-V1 cells were injected subcutaneously into nude mice. Tumor inoculated mice were treated with doxycycline in their daily drinking water (2 g/L) to induce CRT expression. Mice were sacrificed after 15-days treatment and the tumor was removed for experiments. The mRNA expression levels of CRT, VEGF, and GAP43 were confirmed by real-time PCR and were increased after doxycycline treatment. The mRNA expression level was normalized to the internal control HSP60. Each bar of the histogram represents quantified results and is shown as the mean ± SD. Statistical differences were compared with the control level. The expressions of CRT, VEGF and GAP43 were visualized and quantitated by immunofluorescence microscopy. *P < 0.05, **P < 0.01.

The relationship between CRT, VEGF and differentiation of NB was further examined in inducible-CRT stNBV1 cells. After 24 h of tetracycline treatment to induce CRT expression in inducible-CRT stNBV1 cells, the expressions of CRT, VEGF, and GAP43 (a neuronal differentiation marker) were all significantly increased (Fig. 2A).

**Figure 3:**
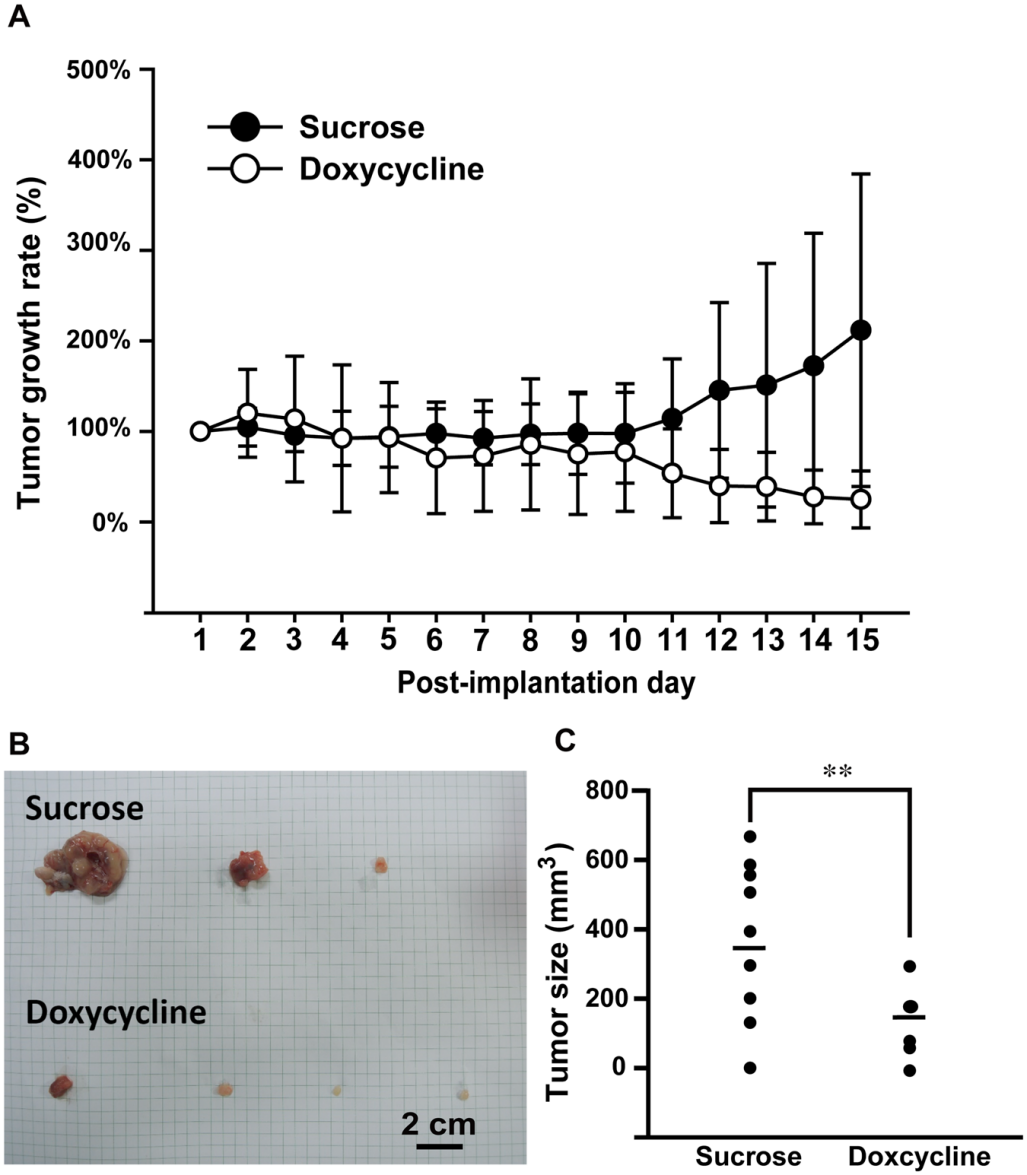
CRT expression suppressed NB tumor growth in mouse xenograft model. (**A**) Inducible-CRT stNB-V1 cells were employed in mouse xenograft model. NB cells inoculated mice were treated with doxycycline in their daily drinking water (2 g/L) to induce CRT expression. The growth of tumor was measured for 15 days. **(B**) Tumor inoculated mice were sacrificed after 15-days treatment and the tumor were dissected to measure the tumor size. Statistical differences were compared with the control level. **P < 0.01.

To further determine the effect of CRT and VEGF on tumor growth, a mouse xenograft model of NB using inducible-CRT stNB-V1 cells was established. NB cells inoculated mice were treated with doxycycline in their daily drinking water (2 g/L) to induce CRT expression. After doxycycline treatment, the xenograft tumors revealed significantly suppressed growth as compared to the control group (Fig. 3A and B). In addition, the mRNA expression levels of CRT, VEGF and GAP43 were all significantly increased in xenograft tumors with doxycycline treatment (Fig. 2B). This result was also confirmed by immunofluorescence microscopy (Fig. 2C).

### VEGF expression was correlated with neuronal differentiation but not angiogenesis in human NB tumors

**Figure 4:**
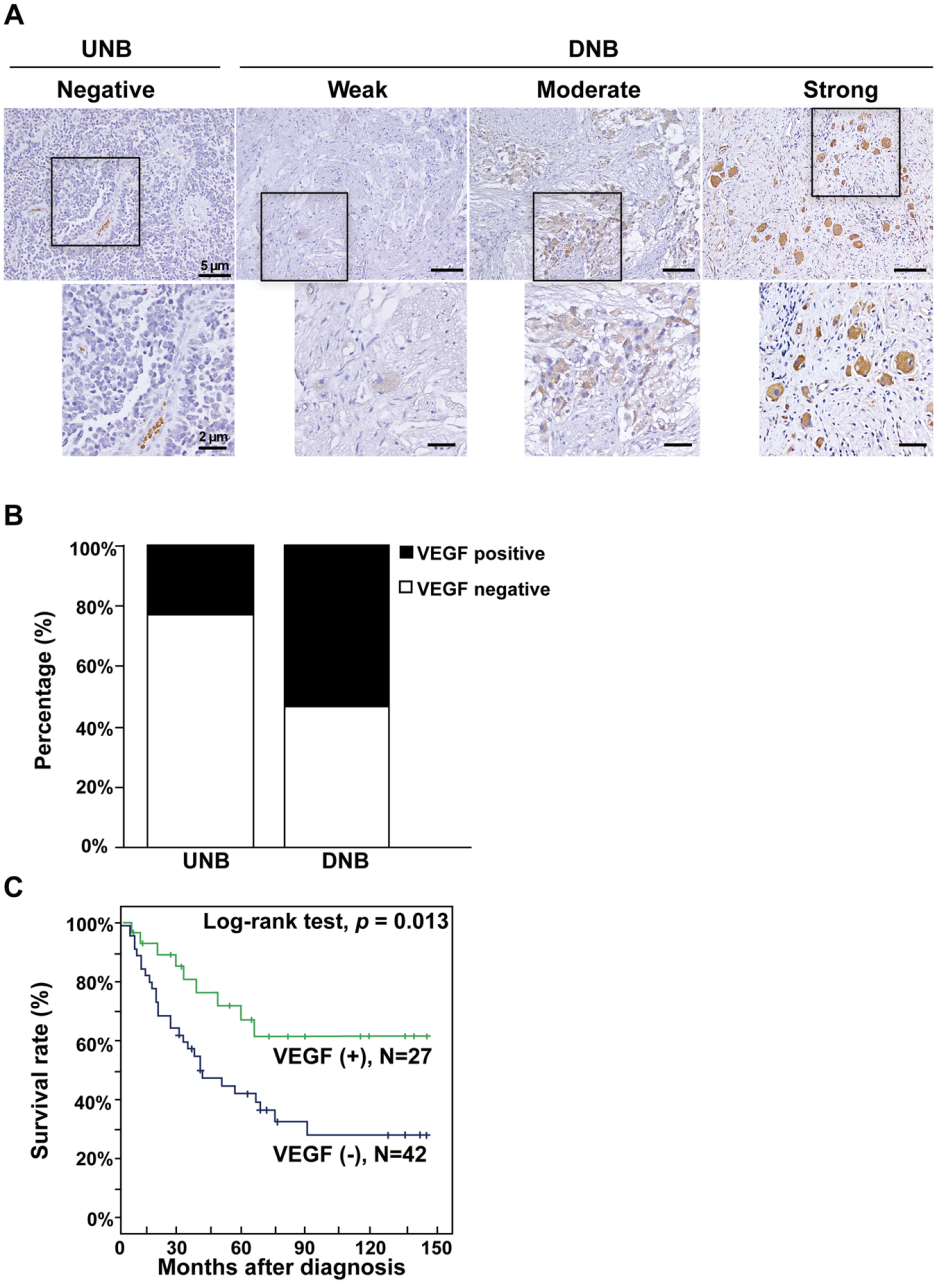
VEGF expression correlated with the histology grades of NB tumors and predicted a favorable clinical outcome of NB patients. (**A**) Immunohistochemical analysis of VEGF expression in tumor tissues of NB patients. Tumors were classified into four categories based on the intensity of VEGF immunostaining: negative, weak, moderate, and strong signals of VEGF. Upper panels shows low magnification images (200× , scale bar, 5 µm) and lower panels indicates the boxed images with high magnification (400× , scale bar, 2 µm). UNB, undifferentiated neuroblastoma. DNB, differentiated neuroblastoma. (**B**) The correlation between VEGF expression level and the differentiation histology of NB tumor was analyzed in 69 human NB tumor samples. Summation of weak, moderate and strong VEGF immunostaining was recorded as “positive”, thereby 23.5% and 54.3% positive VEGF expression were found in UNB and DNB, respectively. (**C**) Kaplan-Meier survival analysis according to the expression of VEGF determined by immunohistochemistry in a cohort of 69 NB patients. NB patients with positive VEGF expression in tumor tissues had a significantly higher 5-year predictive survival rate compared to those patients with negative VEGF expression.

**Figure 5:**
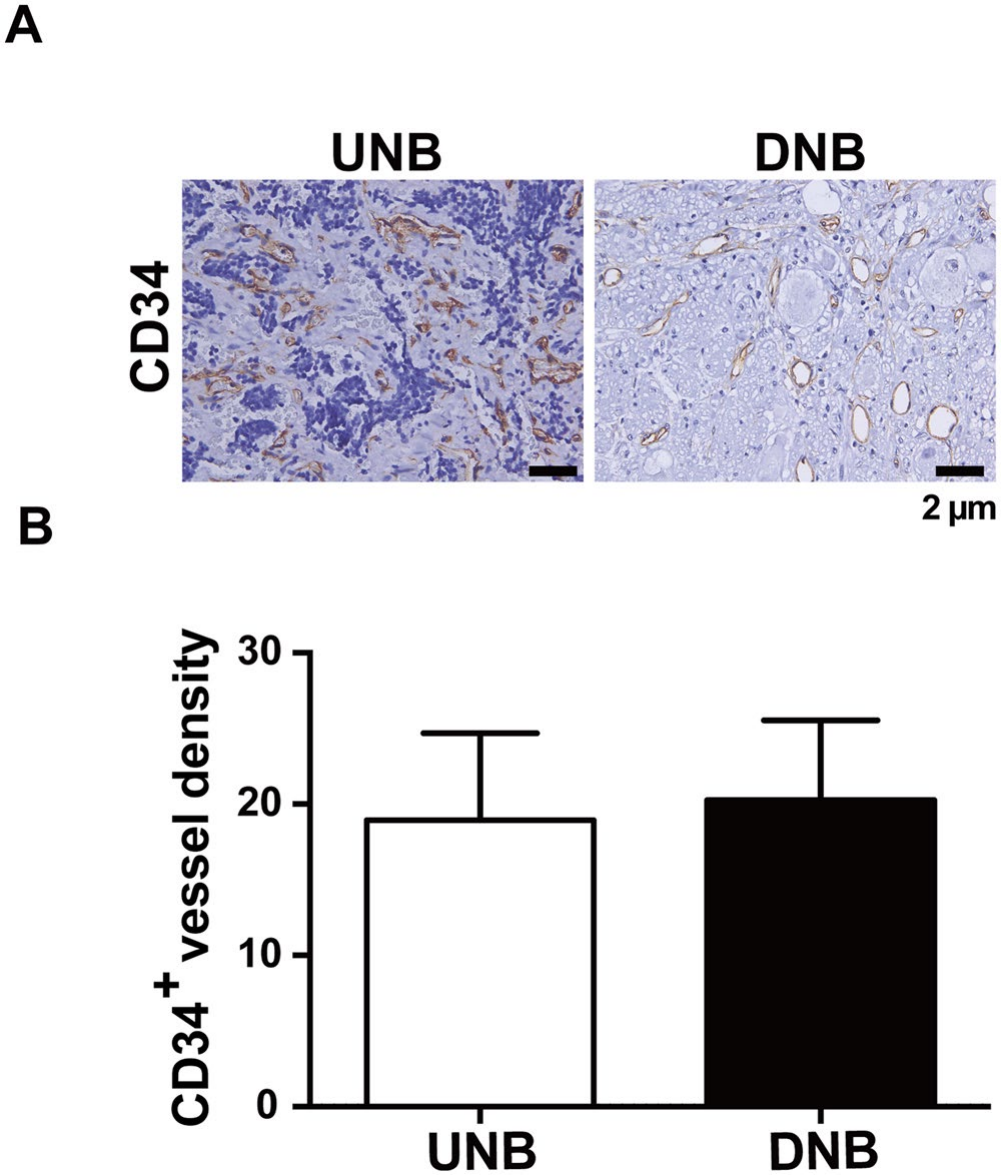
Microvessel density does not correlate with the histology grade of NB tumors. Shown are representative images of microvessels and quantitative data of microvessel density in UNB (N = 21) and DNB (N = 42) tumors staining with blood vessel marker CD34. Data show the mean ± SD. Scale bar, 2 μm.

To investigate if VEGF expression was associated with neuronal differentiation and angiogenesis *in vivo*, the expressions of CRT, VEGF, and blood vessel marker CD34 in 69 human NB tumor tissues were examined by immunohistochemical staining. NB tumors were classified into four categories based on the intensity of VEGF immunostaining: negative, weak, moderate, and strong VEGF signals (Fig. 4A). The results demonstrated that VEGF was more commonly found in NB tumors with more differentiated histology (DNB, 54.3%) than those with undifferentiated histology (UNB, 23.5%) (*P* = 0.013) (Fig. 4B & Table 1). However, there was no correlation between VEGF expression and microvessel density as depicted by CD34 staining (*P* = 0.808). Besides, CD34 immunostaining revealed no significant difference of microvessel densities between human NB tumors with various histology (Fig. 5). Taken together, the study of human NB tumors showed that VEGF expression correlated with neuronal differentiation rather than angiogenesis, suggesting a possible non-angiogenic role for VEGF in promoting neuroblastoma differentiation.

**Table 1:** VEGF expression and clinicopathologic and biologic characteristics of NB.

	Cases	Positive VEGF expression (%)	*P* Value*
Age at diagnosis			
≤1.5 year	22	11 (50.0)	0.290
>1.5 year	47	16 (34.0)	
Sex			
Male	37	13 (35.1)	0.621
Female	32	14 (31.1)	
Clinical Stage			
1, 2, 4S	24	13 (54.2)	0.075
3, 4	45	14 (31.1)	
Histology			
Undifferentiated	34	8 (23.5)	0.013
Differentiated	35	19 (54.3)	
MYCN			
Amplified	15	2 (13.3)	0.034
Non-amplified	54	25 (46.3)	
CRT expression			
Positive	31	19 (61.3)	0.001
Negative	38	8 (21.1)	
Microvessel density			
High	35	13 (37.1)	0.808
Low	34	14 (41.2)	

^*^Chi-square test.

### Clinical significance of VEGF expression in human NB tumors

The clinical significance of VEGF expression in NB was further examined by comparing protein expression of VEGF in various clinicopathologic and biological variables of NB (Table 1). The results showed that positive VEGF protein expression was significantly correlated with CRT expression (*P* = 0.001), in addition to previously mentioned differentiated histology (*P* = 0.013). Furthermore, VEGF expression also showed a strong inverse correlation with *MYCN* amplification, a significant biological marker of poor prognosis of NB (*P* = 0.034). In summary, these results confirmed a positive correlation between VEGF expression and favorable biomarkers including CRT expression, differentiated tumor histology and unamplified *MYCN*.

Kaplan-Meier analysis further revealed that NB patients with positive VEGF expression in tumor tissues had a significantly higher 5-year predictive survival rate as compared to those patients with negative VEGF expression (Fig. 4C; *P* = 0.013, Log-rank test), suggesting that positive VEGF expression could predict a favorable outcome in NB patients.

## Discussion

Converging evidence revealed that NB cells exhibit a capacity of differentiating into neuron-like cells or regression by apoptosis, and hence a benign tumor behavior^20, 21^. In previous studies, we demonstrated that CRT could suppress cell proliferation and enhance cell differentiation^10^. High level of CRT expression in tumor tissues correlates histological grades of differentiation and predicts a favorable prognosis^8^. In addition, CRT can up-regulate the expression and secretion of VEGF protein in various NB cell lines^8, 10^. Blockage of VEGF signaling in turn may suppress neuronal differentiation in CRT-overexpressed NB cells. These lines of evidence suggest that that VEGF is involved in CRT-driven neuronal differentiation of NB. Here we clearly demonstrate that VEGF expression is positively associated with CRT expression as well as neuronal differentiation of NB cells in human tumors, mouse xenografts and cell line models, and may predict a favorable patient outcome. However, VEGF expression is not correlated with the quantitative markers for endothelial cells, indicating an essential role of VEGF in neuronal differentiation rather than angiogenesis of NB.

VEGF is a well-recognized pro-angiogenic factor and a key regulator of physiologic and pathologic angiogenesis^22^. In addition, angiogenesis has been proposed to play a crucial role in regulating NB formation and metastasis^14, 15, 23^. However, there are inconsistent conclusions regarding the role of angiogenesis as well as VEGF expression in the tumor behavior of NB. Some studies showed that high vascular index or over-expression of VEGF was correlated with adverse prognosis of NB patients^24, 25^, whereas other studies revealed that tumor vascularity or VEGF expression was not correlated with prognosis and tumor stage in NB patients^17, 26^. Moreover, it has been shown that there is no significant suppression effect on tumor growth in mouse NB xenografts treated with angiogenesis inhibitor or anti-VEGF antibody^18, 27^. The anti-angiogenesis therapies in NB patients also revealed limited and modest success^3, 28^. In the present study of NB, the microvessel density revealed no significant difference between NB tumors with various histology (Fig. 5 and Table 1), suggesting that angiogenesis is not only related to tumor aggressiveness but also plays an critical role for NB differentiation, which resembles the physiologic events giving rise to the maturation of the vasculature in normal neuronal development. Besides, we did not find a correlation between VEGF expression and vascular index in either the mouse xenografts or human tumor tissues (Table 1). Previous studies mainly evaluated the angiogenic role of VEGF in NB animals or small sample sizes without longitudinal follow-up and got conflict results. In this study, we found no correlation between VEGF and vascular index, but a positive correlation between VEGF and neuronal differentiation of NB. The present study focuses the relationship between NB differentiation and VEGF in tumors of NB patients and found a possible non-angiogenic role of VEGF in NB apart from its angiogenic function. Besides, our longitudinal investigation from a cohort of 69 patients found that positive VEGF expression in tumor tissues had a significantly higher 5-year predictive survival rate. VEGF expression did predict a favorable tumor behavior in NB. These results indicate a particular role of VEGF expression in NB tumor behavior.

It has been shown that VEGF could directly modulate various neuronal functions, including neuronal proliferation, migration, survival, axon guidance, and differentiation in addition to its role in angiogenesis^29, 30^. Evidence has also shown that VEGF up-regulation can promote neuronal differentiation, while VEGF down-regulation inhibits neuronal differentiation both in stroke and hypoxia models^31–34^. Our present studies reveal that VEGF is positively correlated with neuronal differentiation of NB both *in vivo* and *in vitro*, which is compatible with a previous study showing upregulation of VEGF and CEACAM1, a pro-angiogenic factor and mediator of VEGF-driven angiogenesis, during differentiation and maturation of neuroblastic tumors^19^. These findings strongly support the notion that VEGF may affect NB tumor behavior by regulating tumor cell differentiation.

Our previous and present studies demonstrated a strong relationship between CRT and VEGF expression during the differentiation of NB cells^10^. VEGF expression is critical for CRT-driven neuronal differentiation of NB cells^10^. However, how CRT may regulate VEGF expression to affect NB cell differentiation remains unclear. It has been shown that CRT may affect RNA stability by binding to AU-rich element (ARE) in 3′-UTR^6, 35, 36^. By searching the ARE database, VEGF is confirmed to have an ARE on the 3′-UTR region of its mRNA^37^. We then postulate that CRT may regulate VEGF expression by affecting VEGF mRNA stability through the binding of ARE region. Further investigation is needed to verify the mechanism regarding the regulation of VEGF expression by CRT.

Therefore, evaluating VEGF expression in NB could offer complementary prognostic information, which will help clinicians to determine the most proper therapeutic strategies for the NB patients. Studies in the role of VEGF in NB tumorigenesis have unveiled inconsistent results. Both in human and in experimental NB, over-expression of VEGF has been demonstrated and correlated with a high-risk phenotype^11, 12, 38^. In contrast, recent studies have found that VEGF is not related to tumor progression and metastasis in NB^26^. Moreover, it has been shown that there is no tumor growth difference in NB xenograft mice treated with or without anti-VEGF antibody^27^. In our study, for the first time, we demonstrated that positive VEGF expression was significantly correlated with differentiated histology and unamplified MYCN, both of which are favorable prognostic factors. Our present results unequivocally establish VEGF protein expression as a novel independent favorable prognostic factor of NB. Our findings also suggest that anti-angiogenetic or anti-VEGF agents may be inappropriate approaches in managing patients with NB.

In conclusion, this study examines the role of VEGF in regulating NB behavior focusing on angiogenesis and neuronal differentiation both *in vitro* and *in vivo*. The results show that VEGF expression is strongly correlated with neuronal differentiation of NB cells histology in human tumors, mice xenografts and cell line models. Positive VEGF expression is significantly correlated with differentiated tumor histology and normal MYCN status, and hence predicts a favorable patient survival. On the contrary, VEGF as an angiogenesis factor is not correlated with markers of angiogenesis in human tumor tissues of NB. Our findings delineate a novel role of VEGF expression in NB. Instead of enhancing angiogenesis, VEGF might play a critical role in CRT-driven neuronal differentiation of NB. Further studies to decipher the role of VEGF on the regulation of NB differentiation will shed light to the mechanism of tumorigenesis as well as a novel therapeutic strategy to improve the outcome of NB patients in the future.

## Materials and Methods

### Patient cohorts and treatment

A cohort of histologically proven NB patients with complete clinical evaluation and follow-up in National Taiwan University Hospital were enrolled in this study. There were 37 males and 32 females with median age at diagnosis 2.5 years (range 0–11.5). NB tumor specimens were obtained during surgery and immediately frozen in liquid nitrogen. The histologic features of NB were classified into undifferentiated NB (UNB, N = 34) and differentiated NB (DNB, N = 35) according to the International Neuroblastoma Pathology Classification scheme^39^. The clinical stages were determined according to the International NB Staging System (INSS)^40^. *MYCN* amplification was determined by chromogenic *in situ* hybridization^41^. Patients were treated by surgery alone or a combination of multimodal therapy including chemotherapy, radiotherapy, autologous stem cell transplantation, and 13-*cis*-retinoic acid according to the patient’s risk grouping^4^. The clinical evaluation and usage of tumor tissues for this study were approved by the National Taiwan University Hospital Research Ethics Committee. The methods were performed in accordance with the approved guidelines. Written informed consent was obtained from the patients before samples were collected.

### Immunohistochemical staining

A total of 69 tumor specimens collected before chemotherapy were fixed and embedded in paraffin. Tissue sections (5 μm) of tumors were deparaffinized and rehydrated in a routine manner. The expression of CRT, VEGF, and the endothelial cell antigen, CD34, were evaluated using a standard streptavidin–biotin complex immunoperoxidase staining and experimental procedures were performed as described previously^8^. One ganglioneuroma tumor with consistent CRT expression by immunohistochemistry was used as a positive control. Non-immunized rabbit serum was used as a negative control. Tumors with various differentiating histologies were included in each staining. The immunoreactivity of CRT, VEGF, and CD34 were assessed by one pathologist who was blinded to the clinical background of the patients. The immunohistochemical analysis of VEGF was classified into negative, weak (more than 10%), moderate, and strong VEGF signal. Summation of the numbers of samples with weak, moderate and strong VEGF signal was defined as “VEGF positive”. Microvessels staining with CD34 were counted in 400 × field images of three separate intense neovascularized areas, and the mean was calculated as described previously^13^.

### Cell culture

Since constitutive over-expression of CRT leads to NB cell differentiation without proliferation, we utilized stNB-V1 NB cell line and generated an inducible-CRT stNB-V1 cell line by a tetracycline-regulated gene system as described previously^10^. To induce CRT expression, cells were treated with tetracycline (1 μg/mL). Cells were maintained in Dulbecco’s modified Eagle’s medium (DMEM)/high glucose medium containing 10% fetal bovine serum (FBS) and 1% penicillin/streptomycin. The cells were grown in a humidified atmosphere containing 5% CO_2_ and 95% air at 37 °C.

### Mouse xenograft model

The animal experiments were performed after approval from the Institutional Animal Care and Use Committee at National Taiwan University. All methods involved in animal experiments were performed in accordance with relevant guidelines and regulations. For animal models of NB to measure tumorigenity, four-week old female athymic nude mice were housed in pathogen-free conditions and acclimatized for one week. Mice were injected subcutaneously with CRT-inducible stNB-V1 cells. Cells (5 × 10^6^) were suspended in PBS and Matrigel (BD Bioscience) in a 1:1 (v/v) ratio. Tumor-bearing mice were randomized into two groups and were treated with doxycycline in their daily drinking water (2 g/L) (N = 7) or vehicle alone (sucrose) (N = 10) for 15 days. The growth rate of xenograft tumors on animals was measured every day according to the metric measurement of tumor size. Tumor diameters were measured with calipers, and volumes were calculated as width^2^ × length × 0.5. Mice were sacrificed after 15 days, and subcutaneous tumors were surgically excised for further analysis.

### Immunofluorescence analysis

To study CRT, VEGF and GAP43, a neuron specific marker, expression in NB cells, inducible-CRT stNB-V1 cells were cultured on coverslips and stimulated with 1 μg/μL tetracycline for 24 h to induce CRT expression, and then fixed in 4% paraformaldehyde (PFA) for 10 min. After blocking with 5% bovine serum albumin (BSA) for 1 hr, cells were stained with anti-human CRT antibody (Millipore), anti-human VEGF antibody (Santa Cruz), and anti-human GAP43 antibody (Abcam), respectively, at 4 °C overnight. Following the removal of unbound antibodies, cells were stained with Alexa Flour 680-conjugated goat anti-rabbit IgG (Invitrogen) at room temperature for 1 hr. Nuclei were visualized by counterstaining with DAPI. Following extensive washes with PBS, samples were mounted with Fluoromount-GTM (Emsdiasum, Fort Washington, PA, USA). Fluorescence images were acquired using Zeiss AxioPlan 2 fluorescence microscope system. The fluorescence intensity of CRT, VEGF, and GAP43 were quantitated by ImageJ from NIH. Total cells from each viewing area were taken and the fluorescence intensities were quantified. Data were shown as the mean ± SD of the average fluorescence from at least 6 different viewing areas.

To study CRT, VEGF and GAP43 expression in NB xenografts, frozen section slides of tumor samples were first treated with 10% citric acid for 10 mins at 95 °C. After blocking with 5% BSA for 1 h, samples were then incubated with the following primary antibodies, rabbit anti-human CRT antibody (Millipore), rabbit anti-human VEGF antibody (Santa Cruz) or rabbit anti-human GAP43 antibody (Abcam) at 4 °C overnight. Following the removal of unbound antibodies, samples were the incubated with the following secondary antibodies, FITC-conjugated donkey anti-mouse IgG, Alexa Flour 488-conjugated goat anti-rat IgG or Alexa Flour 680-conjugated goat anti-rabbit IgG at room temperature for 1 h. Nuclei were visualized by counterstaining with DAPI for 5 min. Following extensive washed with PBS, samples were mounted with Fluoromount-GTM (Emsdiasum, Fort Washington, PA, USA) and visualized with Zeiss AxioPlan 2 fluorescence microscope.

### RNA isolation and real-time PCR

Total RNA from xenografts and human NB tumors was extracted using the TRIzol reagent (Invitrogen, Carlsbad, CA, USA) following the manufacturer’s instructions. Complementary DNA was synthesized with 1 µg of total RNA using a Toyobo Reverse Transcription -polymerase chain reaction (PCR) kit (Toyobo, Osaka, Japan). The real-time PCR was carried out using a Mini-Opticon real-time detection system (Bio-Rad, Hercules, CA, USA) with the mixture reagent SYBR-Green as the fluorescent dye (Bio-Rad). Gene-specific primers were used and the specificity was confirmed by single melting-curve after real-time PCR. Cycling conditions were 95 °C for 3 min, followed by 30 cycles of 95 °C for 30 s, 60 °C for 30 s, and 72 °C for 30 s. For quantification, the target gene was normalized to the GAPDH to act as an internal control for human NB and heat shock protein 60 (HSP 60) for xenografts. Primers for the real-time PCR were: GAPDH (F-5′-AAG GTG AAG GTC GGA GTC-3′ and R-5′-TGT AGT TGA GGT CAA TGA AGG-3′); HSP60 (F-5′-CA CCG T AA GCC TTT GGT CAT-3′ and R-5′-CTT GAC TGC CAC AAC CTG AA-3′); CRT (F-5′-CC TCC TCT TTG CGT TTC TTG-3′ and R-5′-CAG ACT CCA AGC CTG AGG AC); VEGF (F-5′-GGC ACA CAG GAT GGC TTG AAG-3′ and R-5′-GGC ACA CAG GAT GGC TTG AAG-3′); GAP43 (F-5′-TCC GTC GAC ACA TAA CAA-3′ and R-5′-CAG TAG TGG TGC CTT CTC C-3′).

### Statistical analysis

The statistical analyses were carried out with SPSS 20 for Windows software. The correlation between CRT and VEGF mRNA expression level were analyzed using non-parametric Wilcoxon rank-sum test and Spearman’s correlation test. Other data analyses were performed using one-way analysis of variance (ANOVA), followed by Fisher’s protected least-significant difference (LSD) test (StatView; Abacus Concept, Berkeley, CA, USA). Survival probabilities in various subgroups were estimated using the Kaplan-Meier method, and analyzed by log-rank tests. Statistical significance was set at *P* < 0.05.
